# Pars plana vitrectomy for the repair of primary, inferior rhegmatogenous retinal detachment associated to inferior breaks. A comparison of a 25-gauge versus a 20-gauge system

**DOI:** 10.1007/s00417-012-2059-8

**Published:** 2012-05-17

**Authors:** Roberto dell’Omo, Francesco Barca, H. Stevie Tan, Heico M. Bijl, Sarit Y. Lesnik Oberstein, Marco Mura

**Affiliations:** Department of Ophthalmology, Academic Medical Center, University of Amsterdam, Amsterdam, The Netherlands

**Keywords:** Rhegmatogenous retinal detachment, Small-gauge vitrectomy, Inferior breaks, Sulfur hexafluoride

## Abstract

**Background:**

To compare anatomical, functional outcomes and complications of high-speed 25-gauge (G) pars plana vitrectomy (PPV) versus 20-G PPV for the management of primary inferior rhegmatogenous retinal detachment (RRD) associated to inferior breaks/holes.

**Methods:**

Eighty-five eyes from 85 patients with a minimum follow-up of 3 months were retrospectively evaluated. Forty-one patients underwent 25-G and 44 patients underwent 20-G PPV. All patients underwent PPV with fluid-air exchange, sulfur hexafluoride (SF6) 20 % gas tamponade and laser or cryo retinopexy.

**Results:**

The mean follow-up interval was 6.51(±2.32) and 6.63 (±2.58) months in the 25-G and 20-G groups respectively. Single-operation success rate was 92.7 % for the 25-G group and 81.8 % for the 20-G group (*P* = 0.24). Post-operative hypotony was observed in no case. Redetachment occurred in 3 eyes operated on with 25-G and in 8 eyes operated on with 20-G system. All retinas were attached at final follow-up. Logarithm of the minimum angle of resolution visual acuity significantly improved from 0.69 ± 0.76 to 0.33 ± 0.37 in the 25-G and from 0.47 ± 0.59 to 0.21 ± 0.28 in the 20-G group (*P* = 0.0007 and *P* < 0.0001 respectively).

**Conclusions:**

High-speed PPV and SF6 gas tamponade using either 25-G or 20-G PPV system, yields similar single operation anatomical success rates for the repair of uncomplicated, primary inferior RRDs associated to inferior breaks.

## Introduction

Pars plana vitrectomy (PPV) is gaining popularity for the treatment of primary rhegmatogenous retinal detachment (RRD). In comparison to scleral buckling, PPV offers the possibility of removing vitreoretinal traction, performing a complete drainage of subretinal fluid and precisely identifying and treating the retinal breaks. Further advantages of new transconjunctival sutureless small-gauge (G) systems include reduced inflammation and patient discomfort and shorter recovery time [1–4].

It is generally thought that inferior retinal detachments associated to inferior breaks represent a challenging condition to be managed by PPV without additional scleral buckling for two main reasons: difficulty of completely removing the inferior vitreous, especially in phakic eyes, and difficulty of conventional intraocular tamponade agents of producing a direct tamponade effect on the inferior breaks. High rates of redetachment for inferior detachments treated with standard 20-G PPV have been reported [5].

However, in a large, prospective randomised controlled trial of standard 20-G PPV and gas to repair RRD, inferior retinal breaks were not identified as a risk factor for anatomical failure [6].

Theoretically, disadvantages of PPV for managing inferior breaks or holes might be more evident when using small-gauge equipment because of limitations in ocular rotations due to increased flexibility of instruments and consequent insufficient peripheral vitreous removal and incomplete gas filling.

However, Colyer and associates found no significantly different outcomes using 25-G versus 20-G PPV in pseudophakic inferior RRD [7].

The aim of this study was to compare efficacy and safety of new high-speed 25-G versus 20-G PPV for the repair of primary uncomplicated inferior RRDs with inferior breaks in phakic and pseudophakic eyes.

## Materials and methods

A retrospective, computer-based review of records of 615 consecutive patients who underwent vitreo-retinal surgery for RRD between January 2009 to January 2011 at Academic Medical Centre, University of Amsterdam was performed.

The principles of the Declaration of Helsinki and good clinical practice guidelines were followed. The Institutional Review Board (IRB) at the University of Amsterdam declared that this type of retrospective study waived the need for IRB approval, in accordance with Dutch law on human clinical trials.

Twenty-five G PPV was firstly introduced at our department in 2007, as a new technology initially employed only for eyes with noncomplex conditions such as vitreous hemorrhage and epiretinal membrane.

By the end of 2008, with the availability of high-speed cut rate vitrectomy machines, we extended the indications of small-G systems to more complex cases. Starting from February 2010, all retinal detachments not complicated by PVR grade C or worse were routinely managed by all surgeons using 25-G instruments. The series analyzed in this study includes the eyes that were operated on from January 2009 to January 2011 by the same team of experienced surgeons. The eyes operated on from January 2009 to January 2010 were managed using 20-G PPV; the eyes operated on from February 2010 to January 2011 underwent 25-G PPV. Each surgeon operated a similar number of patients in both groups. All interventions were performed during daytime regular operation hours.

Inclusion criteria were: presence of one or more retinal breaks, or holes between 4 and 8 o’clock associated to detachment involving the lower quadrants. In case of concomitant presence of inferior and superior breaks/holes associated to retinal detachment involving both the lower and the upper quadrants, only the eyes in which the main break was located inferiorly were considered eligible to be included in the study. Location of breaks/holes was ascertained on the basis of the drawings made by each surgeon at the end of the surgical procedure. Therefore intraoperative findings and not the results of pre-operative examinations were considered.

Exclusion criteria included: a follow-up period shorter than 3 months; previous ocular surgery excluding non-complicated cataract extraction; history of any previous vitreoretinal surgical procedure or penetrating ocular trauma; significant ocular comorbidities such as amblyopia, uveitis, glaucoma, proliferative diabetic retinopathy and age-related macular degeneration; acquired or secondary retinoschisis, total RRDs, retinal dialysis, giant retinal tears or proliferative vitreoretinopathy (PVR) of grade C or greater [8].

Pre-operative evaluation consisted of a thorough ophthalmic examination. Logarithm of the minimum angle of resolution (logMAR) visual acuity (VA) was determined by a technician using a standard Early Treatment Diabetic Retinopathy Study (ETDRS) chart at 3 meters implementing the patient’s current spectacle correction. Goldmann tonometry was performed before and the day after operation and at the follow-up visits.

Surgical procedures were performed using the Alcon Constellation (Alcon, Forth Worth, TX, USA) under a BIOM II noncontact panoramic viewing system (Oculus, Wetzlar, Germany). The Constellation exploits a new vitrectomy system with a high-speed cut rate (up to 5,000 cuts per minute, cpm). In all cases, we used cut rates ranging from 2,500 to 5,000 cpm to remove the central vitreous, and a fixed cut rate at 5,000 cpm to remove the peripheral vitreous either using 25-G or 20-G instruments. Vacuum rates ranged from 100 to 650 mmHg and from 100 to 500 mmHg for the 25-G and the 20-G system respectively.

All the eyes underwent core and extensive peripheral vitrectomy over 360 degrees, but shaving of the vitreous base was limited to the areas where retinal defects or suspicious lesions were detected on scleral indentation. All peripheral lesions that resembled breaks or areas of traction received endolaser or external cryo application. Shaving of the vitreous base over 360 degrees was performed in no case. Intra-operative use of perfluorocarbon liquids (PFCL) was at discretion of the operating surgeon.

After air–fluid exchange, SF6 20 % was used as a tamponade in all cases. Patients were asked to avoid supine positioning for 5–days after surgery.

Non-parametric tests (Wilcoxon test, two-tailed Mann–Whitney U test) and parametric tests ( two-tailed *t*-test) were used to assess non-normally and normally distributed data respectively. Significancy of associations was examined using Chi-square and Fisher exact tests.

Logistic regression analysis was conducted to assess evidence of any association between each putative risk factor and failure. Statistical analysis was performed using MedCalc version 11.5.1(MedCalc Software, Mariakerke, Belgium).

## Results

Eighty-five eyes of 85 patients met the inclusion criteria and were selected for final analysis. Of these, 41 had undergone 25-G and 44 had undergone 20-G PPV. Mean (±SD) follow-up duration was 6.51 ± 2.32 and 6.63 ± 2.58) months in the 25-G and 20-G group respectively. Data at 6-month follow-up visit were available for all patients except three in the 25-G group and four in the 20-G group.

The patient demographics are summarized in Table 1.

**Table 1:** Characteristics of the sample

	20-gauge	25-gauge	*P* value
Number of patients	44	41	
Age (years) mean ± SD^a^	60.45 ± 10.0	62.19 ± 10.50	0.43^*^
Male/female	29/15	28/13	0.99^†^
Eye OD^b^/OS^c^	27/17	19/22	0.24^†^
Duration of symptoms (days) median and range	7 (1–60)	7 (1–60)	0.99^‡^
Lens status (baseline)			
Phakic	26	26	0.67^†^
Pseudophakic	18	15	
Lens status (last visit)			
Phakic	20	25	0.17^†^
Pseudophakic	24	16	
Macula status			
On	28	17	0.06^†^
Off	16	24	
Number of breaks/holes (mean ± SD )			
Total	3.15 ± 1.92	2.97 ± 2	0.40^‡^
Inferior	1.61 ± 1.0	1.51 ± 0.81	0.80^‡^
Number of detached quadrants (mean ± SD)	2.29 ± 0.63	2.26 ± 0.67	0.69^*^
Detached quadrants			
1	4	5	
2	23	20	
3	17	16	
Primary anatomical success [*n* (%)]			
Total	36 (81.82)	38 (92.68)	0.24^†^
Phakic	23 (88.46)	25 (96.15)	0.60^**^
Pseudophakic	13(72.22)	13(86.66)	0.41^**^
Re-detachment			
Total	8	3	
Due to PVR^d^ C or worse	5	2	1.0^**^
Reoperations to achieve retinal attachment (number and mean)	11 (1.37)	4 (1.33)	1.0^**^
Follow-up duration (mean, SD, range)	6.63 ± 2.58 (3–21)	6.51 ± 2.32 (3–18)	0.74^‡^
IOP^e^ mmHg^f^ at first day post-op (mean ± SD, range)	20.31 ± 6.99 (10–40)	18.58 ± 7.0 (10–40)	0.15^‡^

^a^SD, standard deviation; ^b^OD, oculus dexter; ^c^OS, oculus sinister; ^d^PVR, proliferative vitreo-retinopathy. ^e^IOP, intraocular pressure; ^f^mmHg, millimiters of mercury; ^g^post-op, post-operative

^*^Two-tailed *t*-test

^†^Chi-square test for independence

^‡^Two-tailed Mann–Whitney U test

^**^Fisher's exact test for independence

There was no pre-operative statistically significant difference for each parameter between the two groups.

Posterior vitreous detachment was diagnosed pre-operatively and confirmed intra-operatively in all cases.

Anatomical success after a single operation was 92.68 % and 81.82 % in the 25-G and in the 20-G groups respectively. The difference was not statistically significant (*P* = 0.24). In both groups, success rate was higher in phakic than in pseudophakic eyes: 96.15 % versus 86.66 % in the 25-G group and 88.46 % versus 72.22 % in the 20-G group. However, such difference was not statistically significant (Chi-square test, *P* = 0.13).

No eye classified as a success after a single operation received secondary vitreoretinal surgery, including retinopexy or intraocular injections, during the follow-up period.

Redetachment occurred in five eyes operated on with 25-G, and in eight eyes operated on with 20-G system; of these, five in the 20-G and two in the 25-G group were attributable to PVR C or worse. All re-detachments occurred within 3 months from the initial operation. All patients presented with an attached retina at the last follow-up visit.

Logistic regression analysis of possible risk factors for anatomical failure after one surgery showed no significant association to age of the patients, duration of symptoms, extension of retinal detachment, total number of breaks/holes, number of inferior breaks/holes, pre-operative lens status and gauge of instruments used for PPV (Table 2).

**Table 2:** Logistic regression analysis of possible risk factors for anatomical failure following primary vitrectomy for inferior, uncomplicated rhegmatogenous retinal detachments

Study factors	OR (95 % CI)	*P* value
Age	1.01 (0.94 to 1.08)	0.769
Duration of symptoms (days)	0.89 (0.76 to 1.04)	0.154
Quadrants of retinal detachment	1.67 (0.46 to 5.99 )	0.425
Total number of retinal defects detected intraoperatively	1.38 (0.75 to 2.53)	0.295
Number of retinal breaks between 4 and 8 hours detected intraoperatively	0.26 (0.05 to 1.33)	0.107
Previous cataract surgery	4.21 (0.97 to 18.24)	0.054
Gauge (25 versus 20)	0.34 (0.07 to 1.72)	0.195

Following surgery, VA significantly improved in both groups (Wilcoxon test, *P* = 0.0007 and *P* < 0.0001 in the 25-G and 20-G groups respectively.

Overall VA gain was more conspicuous in the 20-G group, but during the follow-up period five patients in the 20-G group compared to one in the 25-G underwent cataract extraction (Table 3).

**Table 3:** Pre-operative and post-operative visual acuity

	20-gauge	25-gauge	*P* value^*^
Number of patients	44	41	
Pre-op^a^ BCVA^b^ (logMAR)^c^ [mean ± SD^d^]			
Total	0.47 ± 0.59	0.69 ± 0.76	0.14
Phakic	0.53 ± 0.59	0.73 ± 0.74	0.28
Pseudophakic	0.38 ± 0.59	0.62 ± 0.81	0.56
Post-op^e^ BCVA (logMAR) [mean ± SD]			
Total	0.21 ± 0.28	0.33 ± 0.37	0.057
Phakic	0.24 ± 0.20	0.36 ± 0.39	0.45
Pseudophakic	0.18 ± 0.33	0.29 ± 0.34	0.18

^a^Pre-op, pre-operative; ^b^BCVA, best-corrected visual acuity; ^c^logMAR, logarithm of the minimum angle of resolution; ^d^SD, standard deviation; ^e^post-op, post-operative.

^*^Two-tailed Mann–Whitney U test

The first day after operation, no substantial differences in the amount of tamponade were noted in the two groups: a gas-filling  ≥90 % was recorded in 38 and 42 eyes in the 25-G and the 20-G groups respectively. None of the patients presented with intraocular pressure (IOP) <10 mmHg on applanation tonometry.

Conversely, IOP >22 mmHg was observed in seven eyes in the 25-G group and in 11 eyes in the 20-G group. In all cases, the raised IOP was transient and successfully treated with topical medications.

During the follow-up, two patients in the 25-G group and one patient in the 20-G group developed macular pucker, and one patient in the 20-G group developed a macular hole requiring surgery. In the 25-G group, one case of hemorrhage in the vitreous cavity spontaneously clearing over 3 weeks and one case of choroidal infarct confirmed by angiographic exams and associated to poor VA were noted.

## Discussion

The principle of surgical evolution has always been to make successive procedures less invasive and safer, with quicker recovery and improved outcomes. Over the last years, the advent of small-gauge instruments has sensibly implemented the use of PPV for the management of vitreoretinal pathologies because of its potential advantages such as shorter operating times and faster ocular healing in comparison to standard 20-G vitrectomy [1–4].

In the present study, we decided to focus our attention on uncomplicated inferior detachments originating from inferior breaks/holes to compare the outcomes of 25-G versus 20-G PPV. In fact, small-gauge instruments are commonly deemed not as efficient as 20-G instruments in removing the inferior vitreous, this potentially increasing the chances of failure in presence of inferior breaks/holes and inferior detachment [5].

There are limited published data demonstrating the efficacy of 20-G PPV without additional scleral buckling in the management of inferior RRD with inferior retinal breaks/holes [9–12].

Previous series including phakic and pseudophakic eyes with superior and inferior breaks have reported single anatomical success rate of 74 % [13], 80.5 % [14] and 92.9 % [15] using 25-G PPV. In these series, shaving over 360 degrees was always performed, coupled to 360 degrees laser of the vitreous base in some cases .

A few years ago some of us [16] reported an anatomical success rate of 92.4 % in a series of 131 phakic and pseudophakic eyes with primary RRD associated to superior and inferior retinal breaks/holes operated on with 25-G PPV. In such a series, the surgical approach consisted of peripheral vitrectomy with shaving only in correspondence with the breaks/holes; the location of the breaks/holes did not influence the rate of redetachment.

The anatomical success rate of 92.7 % in the 25-G group of the present series favourably compares with our previous results. The success rate obtained with the high-speed 20-G system is also in line with the results reported for standard 20-G PPV for RRDs involving the inferior quadrants [6, 9–12].

There was no statistically significant difference, in terms of anatomical success after a single operation, in the 25-G group versus the 20-G group. Therefore, in case of inferior RRD, uncomplicated by PVR C or worse and involving one to three quadrants, 25-G PPV does not yield an inferior primary anatomical success rate in comparison to 20-G PPV.

In a retrospective study comparing 25-G to 20-GG PPV, von Fricken and associates [17] reported an anatomic success rate with a single procedure of 90.6 % and 91.8 % respectively. Approximately the same percentage of patients (9.4 % versus 8.2 %) redetached in both groups, and eyes with inferior RRD (41 % of total) were three times more likely to redetach than eyes with superior RRD.

Conversely, in a retrospective comparison of 25-G versus 20-G PPV for repair of primary inferior RRD, Colyer and associates [7] noted that inferior retinal breaks (located between 5 and 7 o’clock) were not associated to higher risk of redetachment. Single operation success rate was 83.3 % for 25-G cases and 89.6 % for 20-G cases.

Population characteristics of our sample differ to some extent from those of the abovementioned series. Similarly to our sample, only eyes with PVR A or B were included, and SF6 was used as endotamponade in the vast majority of cases [7, 17].

However, 55 % of the eyes examined by von Fricken and associates [17] were pseudophakic and 19 presented with high myopia, whereas only pseudophakic eyes were included in the study by Colyer and associates [7].

Furthermore, differently from our series, a conventional vitrectomy system (up to 2,500 cpm) was used, and vitreous base shaving over 360 degrees was applied in all cases, often coupled to 360 laser retinopexy.

It is possible that the high success rate we report in this series is partly attributable to the high-speed vitrectomy system we used to carry out all the surgical procedures. In fact, cut velocity, along with cutter gauge and vacuum rate, are known to be an important factor in determining how much traction is created on the retina during vitrectomy. Furthermore, it has been proposed that high-frequency cutting may be critical in avoiding iatrogenical retinal breaks predisposing to redetachment [18–21].

There is no strong scientific evidence that vitreous base shaving or laser application over 360 degrees may significantly influence the rate of redetachment in primary uncomplicated RRDs. Conversely, both procedures may increase the chance of creating undesired retinal traction and iatrogenic retinal breaks [19–21]. Another inconvenience of shaving is that, even using high-speed vitrectomy devices, the use of PFCL to limit retinal tractions on detached retina and the use of dyes to enhance visualization of vitreous remnants are often mandatory to perform an “as complete as possible” vitrectomy. This makes the surgical procedure significantly longer. Our results might suggest that 360 degrees shaving of the vitreous base and laser over 360 degrees to repair inferior RRDs with certain characteristics (one to three quadrants of detachment and PVR not worse than grade B) could not be as critical as generally thought to influence the anatomical outcome. A less aggressive approach, i.e., the selective identification and treatment of all suspicious lesions by an accurate internal search, could lead to similar success rates while avoiding extra costs and shortening the operating time.

According to our results, shaving of the vitreous base over 360 degrees doesn’t seem to be crucial in preventing the development of severe PVR, which occurred in 8.3 % of the cases operated in this series and has been reported to range between 4.3 % and 16.9 % in previous studies [10–16].

In this series, pseudophakic eyes had a higher rate of redetachment in comparison to phakic eyes in both the 25-G and 20-G groups although the difference was not statistically significant. Worse single surgery anatomical success rates in pseudophakic versus phakic eyes treated with standard 20-G PPV for RRD repair have previously been noted [22, 23]. Also in the series by von Fricken and associates [17], 64 % of the eyes which redetached were pseudophakic.

The decreased anatomical success observed in pseudophakic eyes is probably related to the characteristics of retinal breaks/holes in these eyes. On average, in pseudophakic eyes, the retinal breaks are smaller. Furthermore, especially in presence of lens capsule opacities, the view of the peripheral retina can be unclear. As a result, tiny breaks might be more easily missed, even using enhanced search, leading to surgery failure.

Postoperative hypotony (defined as intraocular pressure <5 mmHg), with or without an accompanying wound leak, has been reported in 0 % to 25 % of cases in retrospective series of 25-gauge PPV [24, 25].

We didn’t find hypotony in any of the cases examined in this series.

Factors that can explain this result are: (a) all cases received gas as endotamponade, the protective influence of which on post-operative hypotony has previously been described [25], and (b) the incomplete removal of peripheral vitreous might have provided an adequate internal plugging for the sutureless sclerotomies.

The retrospective nature and small sample size of this study limit the strength of the conclusions that can be drawn from our data. In line with previous observations, we reiterate that there appears to be no significant difference in outcomes or complications when comparing 25-G to 20-G cohorts [4, 7]. Therefore, the use of 25-G instruments should not be regarded as an inefficient way to treat uncomplicated inferior detachments associated to inferior breaks/holes. Our results also suggest that both 20-G and 25-G PPV can yield similar anatomical success rates in both phakic and pseudophakic eyes, and that shaving of the vitreous base over 360 degrees may be not necessary to achieve these results in uncomplicated inferior RRD.

In summary, the use of 25-G vitrectomy did not significantly reduce surgical efficacy in comparison to standard 20-G vitrectomy when repairing uncomplicated phakic and pseudophakic inferior RRDs associated to inferior breaks or holes.
